# Assessing the competence of midwives to provide care during labor, childbirth and the immediate postpartum period – A cross sectional study in Tigray region, Ethiopia

**DOI:** 10.1371/journal.pone.0206414

**Published:** 2018-10-31

**Authors:** Miruts Goshu, Hagos Godefay, Fantaw Bihonegn, Firew Ayalew, Daniel Haileselassie, Abebe Kebede, Girma Temam, Gebreamlak Gidey

**Affiliations:** 1 Jhpiego, Mekelle, Ethiopia; 2 Tigray Regional Health Bureau, Mekelle, Ethiopia; 3 Jhpiego, Addis Ababa, Ethiopia; 4 Department of Midwifery, College of Health Sciences, Aksum University, Aksum, Ethiopia; University of Michigan Medical School, UNITED STATES

## Abstract

**Background:**

The availability of a skilled birth attendant is widely recognized as a critical factor in reducing maternal and newborn mortality. Competence of maternal healthcare providers directly affects quality of care and health outcomes. This study assessed competence of midwives and associated factors in provision of care during labor, and the immediate postpartum period at public health facilities in Tigray, Ethiopia.

**Methods:**

A cross-sectional study design was employed to collect data through direct observation of the performance of 144 midwives selected from 57 health facilities. Data were collected from January to February 2015 by 12 experienced midwives who were trained on basic emergency obstetric care and had previous experience with data collection. Using a standardized competence checklist, adapted from International confederation of midwives, data collectors interviewed and directly observed the performance of midwives from admission of laboring mothers to six hours after delivery. Multivariable linear regression was used to identify predicators associated with overall clinical competence of midwives.

**Result:**

The mean competence score of midwives was found to be 51%. In multivariable linear regression, male midwifery professionals (p = 0.022), availability of up to date job aids in work place (p = 0.04) and being recognized for improved performance (p = 0.005) were significantly associated with competence of midwives in the provision of care during labor, childbirth and immediate postpartum period.

**Conclusion:**

Competence of midwives was found to be low to provide safe and quality maternity care in the region. Male gender, availability of complete job aids and receiving recognition/awards for better performance were predicted competence. This requires attention and investment from Tigray regional health bureau and health development partners working on maternal and child health. Competence based in-service training, on-the-job mentoring, availing up to dated standard job aids, recognition of high performing midwives are recommended to improve the quality of maternity care in public health facilities of the region. Moreover, affirmative actions including on-the-job training and supervision are needed to improve the competence of female midwives.

## Introduction

The shortage of human resources for health in sub-Saharan African countries severely limits the ability of countries to meet national and global maternal and neonatal health targets and goals. [1, 2] The health worker shortages are further exacerbated by a lack of appropriate knowledge and skills among the existing workforce. [3, 4] Ethiopia has a maternal mortality ratio (MMR) of 412 deaths per 100,000 live births and a neonatal mortality rate of 29 deaths per 1000 live births. [5] Maternal and Child health is at the center of the five year Health Transformational Plan of the Ethiopian government, [6] and its Human Resources for Health strategy has set a target to train 8,635 midwives by the year 2015. [7] The country has also set a target to reduce its MMR to 199 per 100,000 live births by the year 2020. [6] A study conducted in the Tigray region of the country estimated that the MMR for the region was 266 deaths per 100,000 live births [8], and skilled birth attendance coverage was reported to be 59%. [5] One of the factors contributing to the high level of maternal and perinatal mortality and morbidity is poor quality of care. [9] Without ensuring the availability of adequate numbers of competent providers at health facilities, other strategies designed to reduce maternal, neonatal mortality and morbidity cannot be effective. [10] In Ethiopia in general and in the Tigray region in particular, there is lack of evidence on the competence of midwives. This study explored the following research questions: 1) How competent are midwives in providing care during labor, childbirth and the immediate postpartum period in public health facilities? and 2) What are the factors associated with competence of midwives in provision of care during labor, and the immediate postpartum period at public health facilities?

## Materials and methods

### Study design and sample size

The study employed a cross-sectional design using direct observation of midwives while performing labor and delivery, and postpartum care in public health facilities in Tigray region. There were a total of 228 health facilities (14 hospitals and 214 health centers) and 662 midwives in the region during the study period. An optimum sample size was calculated to provide regionally representative information using the following sample size calculation assumptions: 95% level of confidence, a design effect of 1.2, proxy reference values of 51.8% mean competence score of midwives, with standard deviation value of 15.3 [11], 5% of relative error from percentage mean competence score (equivalent to 2.58 margin of error) and adding 10% non-response rate. Based on these statistical parameters, an estimated sample of 144 midwives was calculated as outlined below:

Minimum sample size required $(n)=\frac{Z^{2}{}_{1-\alpha }\left(SD^{2}\right)Deff}{d^{2}}=\frac{1.96^{2}X15.3^{2}X1.2}{2.59^{2}}=161$ Where:

Z_1-α_ = 95% confidence interval with critical value of 1.96
SD = Standard deviation with value 15.3
Deff = Design effect value of 1.2
d = assumed margin of error with value 2.59 (or 5% of relative error from the mean score or 51.8),
N = total number of midwives working in health facilities of Tigray region (N = 662).

Since ratio of n/N is > 0.5 in the region, the sample required adjustment using the formula;

n/ (1+n/N) and 10% nonresponse rate. Hence, the final adjusted sample size for inclusion in the study was 144 midwives.

Data from the Tigray regional health bureau suggested that an average of ten midwives was employed at each public hospital, and three midwives at each heath center. Considering resources and the total number of midwives available, a sample of four midwives per public hospital and 2 midwives per health center was determined to be optimal for observation and interview. Accordingly, four midwives were randomly selected in each hospital and two midwives randomly selected in each sampled health center for observation. In order to get heterogeneous information, we divided the total estimated sample of 144 midwives into hospitals and health centers using a proportional allocation technique. This resulted in a sample of 52 midwives in 13 hospitals and 92 midwives in 46 randomly selected health centers in the study. Thirteen out of the 14 existing hospitals were included; one hospital was excluded because of geographic inaccessibly. The 46 sample health centers were randomly selected from a list of total 214 health centers. Two health centers from the sample 46 health centers were omitted from the study because there were no laboring mothers during the data collection period.Assessors invited randomly selected midwives to observe their performance when they were providing labor and delivery services. Assessors did not observe midwives who were not selected randomly even if she or he provided labor and delivery care. Additional midwives were observed from hospitals to replace midwives working in health centers because of the lack laboring mothers during data collection time at these health centers.

### Instruments

The contents of the midwifery competence assessment checklist were adapted from the essential competencies for basic midwifery practice defined by the International Confederation of Midwives (ICM). [12] Twelve competence areas were included in the study to observe midwives while attending laboring mothers. Each competence domain had a description explaining what the competence criterion includes, which guided assessors to give appropriate scores. The competencies included 1) performing rapid initial evaluation at first contact; 2) introduction & history taking; 3) perform physical examination; 4) use of partograph to monitor labor progress; 5) assist the woman to have a safe and clean birth; 6) providing immediate postpartum care; 7) clinical judgment/ decision-making in providing care; 8) responding to problems/ irregularities if any; 9) infection prevention; 10) communication; 11) organization, efficiency and team work; and 12) humanistic qualities/ professionalism. For each competence item, assessors provided a rating ranging from 1 to 9 indicating unsatisfactory (1 to 3) when the provider did not perform the task or attempted to perform the task but was below expectation; satisfactory (4 to 6) when the provider performed the task correctly & met expectations/minimum standards, and superior (7 to 9) when the performance was above expectation and the provider could be considered expert or a master, able to teach others. Midwives’ socio-demographic characteristics were included in the questionnaire, as were questions related to the availability of relevant trainings, practice-based learnings, supervisory support, availability of standard and up-to dated job aids, recognition/awards, infection prevention equipment and supplies, medical equipment (such as sphygmomanometer, thermometer, delivery kits, episiotomy sets, vacuum, bag and mask for newborn resuscitation, medical supplies (like glove, sutures), emergency medications and equipment (such as utero-tonics, magnesium sulphate, IV solutions, needle, syringe), records and forms (Partograph, delivery log or register), a toilet in the delivery area, and water in delivery area and new born care unit. The response options for the availability of trainings, practice based learnings opportunities; supportive supervision, job aids, equipment, supplies and infrastructure were “yes” or “no”.

### Data collection

Twelve basic emergency obstetric care (BEmONC) trained expert midwives who had previously been selected by the regional health bureau to assess midwifery competence of graduating students in the region for another study were recruited to conduct the data collection. Before deployment, data collectors were trained for three days on how to obtain consent, observation approaches, interviewing skills, and maintaining data confidentiality. During the training they pre-tested the tools in four health centers and demonstrated their performance in conducting the competence assessment of midwives using role play.

Data were collected by six teams with two data collectors and one supervisor in each team. Each team of data collectors was immediately deployed to assigned health facilities after the training. Data were collected from January to February 2015. The data collection team spent a maximum of two days at each health center, and a maximum of 4 days at each hospital. Four co-investigators and two faculty members from Mekelle University supervised the field data collection process, verifying that data collectors were recording data appropriately. The study team first met the person in charge of each health facility and explained the purpose of the study, presented letters of approval from the Regional Health Bureau and answered questions before beginning any data collection. Study team members then met all eligible participants at each facility and explained the study, and obtained verbal consent.

Observations were documented using a standardized checklist on the various domains of competencies. Midwives were directly observed while providing care during labor, delivery and in the first 6 hours after birth. One labor and delivery procedure was observed per midwife provided that the woman went through all stages of labor and delivery. If observation was incomplete (that is the provider did not complete labor and delivery observation), data collectors observed the performance of the midwife during her/his care of the next laboring woman.

After the observation, midwives were interviewed using a structured questionnaire to capture socio-demographic characteristics and availability of an enabling environment for maternity care. The interview took place at a time and place that was convenient for the midwives and lasted approximately 15 minutes.

### Data management and analysis

Epi-data version 2.0.2.13 was used for data entry and SPSS version 23 was used for further statistical analysis. Descriptive analysis performed to calculate frequencies and mean competence percentages. Linear regression analyses was used to explore relationship of providers’ mean competence score and predictors, including: background characteristics (gender, age, education level, education program attended, facility type and experience), technical update training in the last two years, equipment and supplies, availability of standard and up-to date job aids, availability of forms and records, toilet, water, newborn care unit, regular supportive supervision, regular seminar/case presentation and award/recognition. Predictors in the bivariate linear regression models were selected as candidate predictors for Multivariable linear regression using p-value of ≥0.25. Before fitting the final model, regression model assumptions including multicollinearity, normality, homoscedasticity and potential outliers were checked, Based on these criterion, thirteen predictors were included in the final multivariable regression analysis. A p-value of less than 0.05 was used for making statistical significance decision.

### Ethical consideration

The study protocol was approved by the Johns Hopkins School of Public Health Institutional Review Board (IRB#6118). A letter of support was sent from the Tigray regional health bureau to each participating health facility. Informed oral consent was obtained from all participants (midwives and laboring mothers or their kinship) after the aim of the study was explained–the consent form was translated to the local language, Tigrinya. The steps taken to preserve confidentiality of information includes: de-identification of 07X aster, able to teach others bureau to assess competence of midwives before graduation of students in the region. Personal information, completed assessment study tools were kept in locked cabinet and electronic datasets were protected with a password.

## Result

### Socio demographic characteristics of midwives

The response rate of participants was 100%. Majority (70.8%) of study participants were female, and had a diploma/level IV education (73.6%). The mean age of the participants was 33.9 ± 9.5 years, and 41.7% had five or more service years. Most (56.9%) were working at a health center (Table 1).

**Table 1:** Characteristics of midwives, Tigray region, Ethiopia (N = 144).

Variable	Number	%	Mean	SD*	Range (Min, Max)
**Facility type**					
*Health Center*	*82*	*56*.*9*			
*Hospital*	*62*	*43*.*1*			
**Gender**					
*Male*	*42*	*29*.*2*			
*Female*	*102*	*70*.*8*			
**Education level**					
*BSc*. *Degree/Bachelor/*	*38*	*26*.*4*			
*Diploma/level IV*	*106*	*73*.*6*			
**Service year**					
*less than one year*	*29*	*20*.*1*			
*1 to<5year*	*55*	*38*.*2*			
*> = 5years*	*60*	*41*.*7*			
**Education program**					
*Generic*	*67*	*46*.*5*			
*Upgrade*	*77*	*53*.*5*			
**Average deliveries per midwife per day**			2.5	1.3	1, 5
**Age in years**			33.9	9.5	33 (20, 53)

*SD: Standard Deviation

### Midwives response about their working environment

Majority of the midwives reported that there was regular supportive supervision and on-the-job learning which included case presentations, seminars and bedside rounds. Only 38.2% of the midwives reported that they had adequate equipment and supplies, 42.4% had a toilet in the delivery room, 51.2% had water in the delivery room and 28.5% had received awards for good performance (Table 2).

**Table 2:** Midwives’ positive responses on characteristics of their work environment, Tigray region, Ethiopia.

Variables	Number (Yes)	Percent (%)
Availability of complete job aids	55	38.2
Availability of medical equipment and supplies	75	52.1
Availability of forms and records	71	49.3
Availability of a toilet in the delivery room	61	42.4
Availability of water in the delivery room	74	51.4
Availability of a new born care unit	78	54.2
Technical update training offered within the previous two years	98	68.1
Availability of regular supportive supervision	123	85.4
Availability of on-the-job learning opportunity (case presentations, seminars and rounds)	118	81.9
Received recognition/awards for good performance	41	28.5

### Overall and specific mean percentage competencies of midwives

The overall mean competence score reflecting average competence across 11 areas was 51%. One competence area (responding to problems/irregularities) was excluded because very few midwives were observed providing this service. The mean score was comparable for both facility type and education level, however, being male was positively associated with having a higher competency score. The midwives scored highest in organization, efficiency and team work (58%) followed by humanistic qualities/professionalism (57%). Their competence was lowest in conducting rapid initial evaluation at first contact (41%) followed by use of partograph to monitor labor progress (43%). Being male was associated with achievement of higher scores in specific competencies including performing rapid initial evaluation at first contact of women, introduction and history taking, perform physical examination, use of partograph to monitor labor progression, assist the women during labor to have safe and clean delivery, provide immediate postpartum care and clinical judgment/decision making in providing care (Table 3)

**Table 3:** Overall and specific mean percentage competence scores of study participants by gender, type of facility and level of education. Tigray region, Ethiopia.

Type of competence	Percentage Mean competence	Gender			Type of facility			Level of education		
		Male	Female	P-value*	Hospital	Health Center	P-value*	Bachelor	Diploma	P-value*
Perform Rapid initial Evaluation at first contact	41.0	48.9	37.7	**0.001**	42.1	40.1	0.513	46.4	39.0	**0.028**
Introduction and history taking	45.0	54.4	41.1	**0.000**	46.1	44.2	0.523	51.8	42.6	**0.007**
Perform physical examination	52.0	56.7	49.4	**0.019**	50.3	52.4	0.474	54.1	50.6	0.271
Use partograph to monitor labor progression	43.0	51.6	40.6	**0.005**	44.1	43.4	0.861	50.8	41.3	**0.019**
Assist the women to have a safe and clean birth	55.0	59.8	53.2	**0.022**	52.9	56.9	0.125	55.6	55.0	0.860
Provide immediate postpartum care	50.0	55.8	47.4	**0.001**	50.0	49.9	0.952	50.6	49.7	0.724
Clinical judgment in providing care	53.0	60.3	50.0	**0.000**	51.7	54.1	0.335	55.2	52.2	0.299
Responding to problems if any (n = 28)	54.0	60.3	51.9	0.324	51.0	58.6	0.317	55.6	53.2	0.771
Infection prevention	51.0	52.1	50.4	0.571	50.7	51.1	0.893	51.2	50.9	0.914
Communication	52.0	60.6	55.9	0.100	56.1	58.1	0.417	57.3	57.2	0.979
Organization, efficiency and teamwork	58.0	61.3	57.0	0.068	57.3	58.9	0.473	58.2	58.3	0.970
Humanistic qualities/ professionalism	57.0	56.9	57.2	0.917	54.7	58.9	0.115	55.0	57.9	0.345
**Overall mean competence**	51.0	56.4	49.0	**0.001**	50.6	51.6	0.607	53.6	50.3	0.164

*Independent sample t-test

### Factors associated with overall mean competence of midwives

The bivariate linear regression model found that the mean competence score of midwives was significantly associated with male gender, experience, availability of complete job aids, availability of water, conducting regular on-the-job learning and recognition for good performance. Three variables: male gender (p = 0.022), availability of complete job aids (p = 0.04) and recognition for good performance (p = 0.005) remained significant in a multivariable linear regression (Table 4).

**Table 4:** Bivariate and multivariate linear regression analysis of factors associated with competence score of Midwives. Tigray region Ethiopia.

Variable	Bivariate linear regression		Multivariate linear regression	
	Coefficient (95%CI)	P-Value	Coefficient (95%CI)	P-value
Male gender (reference: Female)	7.10(2.90, 11.3)	0.001*	5.45 (0.79, 10.1)	0.022*
Age (in years)	-0.20 (-0.40, 0.01)	0.062	-0.201 (-0.57, 0.17)	0.28
Education level (reference : Diploma)	2.80 (-1.63, 7.27)	0.212	2.31 (-2.57, 7.18)	0.35
Experience in months	-0.03 (-0.05, -0.004)	0.021*	-0.01 (-0.04, 0.012)	0.31
Education program (reference : upgrade)	2.55 (-1.39, 6.48)	0.20	-4.22 (-10.96, 2.52)	0.22
Availability of complete job aid (reference : No)	6.21 (2.28, 10.13)	0.002*	4.38 (0.198, 8.57)	0.04*
Availability of forms and records (reference : No)	0.67 (-3.27, 4.62)	0.74	1.77 (-2.32, 5.87)	0.39
Availability of toilet in delivery room (reference : No)	2.76 (-1.20, 6.73)	0.17	-1.40 (-5.70, 2.91)	0.52
Availability of Water in delivery room (reference : No)	4.32 (0.44, 8.20)	0.03*	2.77 (-1.21, 6.75)	0.17
Availability of new born unit (reference : No)	3.79 (-0.12, 7.70)	0.057	0.48 (-3.65, 4.60)	0.82
Availability of regular supportive supervision (reference : No)	4.71 (-0.82, 10.25)	0.09	3.26 (-2.18, 8.71)	0.24
Availability of on job learning (case presentation, seminars and rounds) (reference : No)	6.35 (1.33, 11.37)	0.013*	3.10 (-1.85, 8.06)	0.22
Received recognition/awards for better performance (reference : No)	6.31 (2.07,10.55)	0.004*	5.75 (1.76, 9.75)	0.005*

*Variables significantly associated with mean competence of midwives (P-Value <0.05) in bivariate and multivariate analysis.

## Discussion

Ensuring the equitable provision of quality maternal health services is one of the essential strategies to reduce maternal and neonatal morbidity and mortality in Ethiopia. [5] Our study found that the overall mean competence score of midwives working in Tigray public health facilities was low, which is consistent with other studies [11, 13–17] conducted in low resource settings. This may be a reflection of approaches that on shorter-term solutions that aim to increase the numbers of providers [18] rather than a focus on ensuring that they have the required competencies. [3]. This low competence score of midwives in our study could be attributed to the students’ perceived poor quality of health care providers’ pre service education in Ethiopia [11,19] including gaps in availability of quality and adequacy of teachers, educational resources and the practical sites. In a study conducted in Addis Ababa, insufficient in-service and pre-service trainings were also identified as barriers to providing quality basic emergency obstetric and neonatal care. [20] The poor competence of midwives demonstrated in our study underscores the urgent need to refocus on both pre service and in-service training of midwives. Major international consortiums and initiatives, including the United Nations Global Strategy for Women’s and Children’s Health [21] and the United States of America government Global Health Initiative [22] have identified health workforce capacity building as a key element in strengthening health systems and for achieving improved health outcomes, particularly for women and children. Strategies common to programs for enhancing health workforce capacity include workforce training, improved supervision, and the use of monetary and non-monetary incentives. [23–27].

Female midwives scored lower than their male counterpart in certain areas. These results are consistent with previous studies conducted in Ethiopia looking at graduating midwifery [11] and anesthesia students.[19] These persistent findings of lower competence of female health care providers requires further investigation to identify challenges faced by female health care providers in gaining and maintaining the required skills and competencies.

We found that the availability of job aids was significantly associated with improved competence of midwives. Our findings were consistent with studies conducted in Benin and Uganda in which interventions focused on job aids were linked with positive outcomes on providers’ practices. [28–29] Moreover, recent evidence shows that job aids can serve health care providers as an acceptable, low-cost alternative to improve their performance when combined with minimal training and supervision. [30–34] In line with this finding, it is important for the Tigray regional health bureau and its stakeholders to avail relevant job aids in all health facilities to guide midwives’ performance.

The study also found that recognition for good performance is significantly associated with higher competence of midwives. This finding is consistent with a Ugandan study [29] which found that recognition of health care providers was associated with improved performance in counselling. Therefore, performance appraisal linked with recognition needs to be strengthened as a means to improve performance of midwives in provision of maternity care in the region.

One limitation of the study is that the Hawthorne effect–the performance of the midwives may have been altered because they were aware of being observed. Another limitation of the study could be lack of national benchmarks to define the competence level of midwives. However, we believe that the findings are generalizable to the Tigray region, and provide useful insights for policy makers and other stakeholders.

## Conclusion

The competence of midwives working in public health facilities in Tigray was found to be low, thus suggesting potential concerns regarding the quality of maternal and child health services in the region. Male midwives, availability of complete job aids and receiving recognition for good performance predicted competence. Urgent attention and more investment from the Ethiopian Ministry of health, the Tigray regional health bureau and health development partners working in improving maternal care is required to attain the 2015–2020 health sector transformation plan [5] and sustainable development Goal (SDG) target of reducing MMR to less than 70/100,000 [35]. Competence based in-service training, on-the-job mentoring, availing up to dated standard job aids, and recognition of high performing midwives is recommended. Moreover, affirmative actions including on-the-job training and supervision are needed to improve the competence of female midwives.

## Supporting information

S1 File Recruitment & oral consent script #1 (Tigrigna language translation). (PDF)

S2 File Recruitment & oral consent script #2 (Tigrigna language translation). (PDF)

S3 File Recruitment & oral consent script #1 to be read aloud to midwives. (PDF)

S4 File Recruitment & oral consent script #2 to be read aloud to client (women in labor and delivery). (PDF)

S5 File Performance assessment of midwives in provision of care during labor, childbirth, and immediate postpartum period in Tigray and Amhara regions, Ethiopia. (PDF)

S1 Dataset SPSS data Competence Assessement of Midwives in Tigray health facilities. (SAV)
