# Supplementary material for: Assessing the competence of midwives to provide care during labor, childbirth and the immediate postpartum period – A cross sectional study in Tigray region, Ethiopia
Source: PLoS One. 2018 Oct 31;13(10):e0206414. doi: 10.1371/journal.pone.0206414 (PMC6209306; doi:10.1371/journal.pone.0206414)
Supplement: S1 File — (PDF) [file pone.0206414.s001.pdf]

EXEMPT
HSPH Institutional Review Board
IRB #6118
Exempt Determination Date: 04Dec14
Version No.: 1

RECRUITMENT & ORAL CONSENT SCRIPT #1

TO BE READ ALLOUD TO MIDWIVES

(TIGRIGNA LANGUAGE TRANSALTION)

RECRUITMENT SCRIPT

ፅሑፍ ምልመላን ናይ ቃል መረጋገፂ ስምምዕ ተሳትፎ ቁፅሪ 1

ን "መዋለድቲ" "ሓፍ" ብዝበለ ድምፂ ዝንበብ

ፅሑፍ ምለመላ ምልከታ

ከመይ ቀኒኽን፡ ሽመይ ________________ ይብሓል፡፡ ብዝአባ ዝኾነ መፅናዕቲ ምሳኩም ክዘራረብ ደሊየ ኔረ፡፡

አብ ፕሮጀክት ሓላዋ ጥዕና መምርሒ ምልማዕ ኃይሊሰብ ከም "መማኸሪ" ኮይነ ዝሰርሕ እንተኸወን፡ እዚ መፅናዕቲ እዚ "መዋለድቲ" (ንአፈፃፀጽማ ስራሖም ንዝትንክፉ ፍልጠትን፣ አፈፃፀማ ስራሕን አረዳድአ ኩነታት ጠመተን ዝመሳሰሉ ዋኒናት ብዝሕውስ መልክዕ) አብ ሰራሖም ንዝሕልዎም ኩነታ አፈፃፀማ ብዝሐሸ መንገዲ ንምርዳእ ወይካአ ንምግምጋም ተሓሲቡ ምስ ክልላዊ ቢሮ ሓለዋ ጥዕና (ምምሕድዳር ክልል አምሐራን) ክልላዊ ቢሮ ሓለዋ ጥዕና (ምምሕድዳር ክልል ትግራይን) ብምትሕብባር ዝተከየደ/ዝተዳለወ መፅናዕቲ እዩ፡፡

"ምሳኽን ንቁሩብ ደቓይቅ ክዘራረብ ሰለዝደለኹ፡ ሕጂ ሓንሳብ ጊዜ ወሲድክን ክትድምፃኒ ፍቓደኛ ዲኽን?"

["አይኾንኹን" እንድሕር ኮይኑ] "አብ ካልእ ጊዜ ንከዛረብክን/ኩም ትፈቅዳለይ/ዱለይ ዶ?"

["አይኾንኹን" እንድሕር ኮይኑ] "ፅቡቕ፡ ንዝሓብክና/ኩምሚ ጊዜ የመስግን፤ ድሐን ዋዕላ/ሉ!"

እቲ መቕረባይ ቃል-ሕቶ ነቲ ተቃውሞ አብቲ መዝገብ ምልመላ ብፅሑፍ የስፍር፡፡

[“እወ” እንድሕር ኮይኑ] “የቀንየለይ፣ እምበአርከስ ብዝኣባ ክንስርሕ ዝተለምናዮም ነገራት ተወሳኺ ሓበሬታ ክሕበክን/ኩም”

“ንትሳተፋሉ/ፉሉ ከይዲ ሕማም ሕርሲን ሕክምና ሕርሲን/ወሊድን ከይዲ ሕክምና ኽንኽን ሕርሲን ንዕዘብ ኮይኑ፣(ንአፈፃፀጽማ ስራሖም ንዝትንክፉ ፍልጠትን፣ አፈፃፀማ ስራሕን አረዳድአ ኩነታት ጠመተን ከሙእውን አቅርቦት መሳሪሕታትን መሰረተ ልምዓትን ዝመሳሰሉ ዋኒናት ብዝሕውስ መልክዕ) አፈፃፀማ ስራሕክን/ኩም ዝመልከቱ ገለ ገለ ሕቶታት እውን ክንሐተክን/ከም ኢና።

“ሽምክን/ኩምን ወይካአ ሽም ዘመድንክን/ኩምን ዘይምዝገብ እንተኸውን፣ ብዝኣባ እዚ ዋኒን እዚ ዝርከቡ ሐበሬታት ብሙሉኦም ብምሽጥር ዝተሓዙ ኮይኖም አብዚ ወይከአ አብ ካልኦት ትካላት ሐለዋ ጥዕና ንዝርከቡ ወይከአ ንዝሰርሑ ሰባት አይግለፁ።

“አብቲ መጽናዕቲ ናይ ግድን ክትሳተፉ/ፉ አይትግዳዳን/ዱን። ንምስታፍ ብዘይምድላይኽን/ኹም ንስራሕክን/ኩም ወይካአ ንትረክብዎም ካልኦት ጥቅማጥቅሚታት አይትንክፍን ወይካኣ አይተኽናቅፍን።”

“ብዝኣባ እዚ መፅናዕቲ እዚ ዝርዝር ሓበሬታ ናይምፍላጥ ድሌት አለክን/ኩም ዶ?”

[“የብለይን” እንድሕር ኮይኑ] “ፅቡቕ ፣ ንዝሓብክና/ኩምና ጊዜ ነመስገን ፤ድሓን ዋዕላ/ሉ!”

“እቲ መቕረባይ ቃል-ሕቶ ነቲ ተቃውሞን አብ መዝገብ ምልመላ ተሓኪምቲ/ ተገልገልቲ ብፅሑፍ የሰፍር”

[“እወ” እንድሕር ኮይኑ] “ፅቡቅ ፣ እምበአርከስ ብዝኣባ እዚ መፅናዕቲ እዚ ዝርዝር ሐበሬታ ክሕበክን/ኩም። አብዚ መፅናዕቲ እዚ ንምስታፍ እንድሕር ተስማሚዕክን/ኩም ክትነግራኒ/ክትነግሩኒ ትክዕላ/ሉ”

[ብዚ መሰረት‘ውን እቲ መቕረባይ ቃል -ሕቶ ቅልጥፍ ኢሉ ወይካአ ብእዋኑ ነቲ ከይዲ አፈፃፅማ (ናይ ቃል) መረጋገፂ ስምምዕ ተሳትፎ አብ ቀፃሊ ገፅ ምድላው/ምምላዕ ይጀምር]

IRB #6118
Exempt Determination Date: 04Dec14
Version No.: 1

ORAL CONSENT SCRIPT #1

ፅሑፍ ምልመላን ናይ ቃል መረጋገፂ ስምምዕ ተሳትፎ ቁፅሪ 1

ን "መዋለድቲ" "ሓፍ" ብዝበለ ድምፂ ዝንበብ

ፅሑፍ ምለመላ ምልከታ

ዓርስቲ መፅናዕቲ ፡ ኣብ ኢትዮጵያ ክልላት ትግራይን ኣምሓራን ኣብዝርከቡ ትካላት ሓለዋ ጥዕና ኣብ ዘፈረ-ግልጋሎታት ሕማም-ሕርሲን፣ህክምና-ህርሲን/ወሊድን ከይዲ ሕክምና ህርሲን/ወሊድን ንዝሰርሖ "መዋለድቲ" ዝምላዕ ሰነድ መገምገሚ ብቅዓት

ዋና ኪዒላ መፅናዕቲ/መርማሪ ፡- ዶ/ር ያንግ ሚ ኪም

ቁፅሪ ኣይኣርቢ ፡ 6118

ዕላማ

ኣብዚ መፅናዕቲ/ምርምር እዚ ንክትሳተፉ/ፋ ስለዝተኣደምክን/ኩም፣ ብዝኣባ እቲ መፅናዕቲ ቁሩብ መብርሒ ክሕበክን/ኩም ይፈቱ፡፡

ነቲ መፅናዕቲ ዘካይዶ ኣካል

እዚ መፅናዕቲ እዚ ዝካየድ ዘሎ ኣብ ኢትዮጵያ ብዝርከብ ፕሮጀክት ሓላዋ ጥዕና መምርሒ ምልማዕ ኃይሊ ሰብ እንተኸውን ፤ እዚ ፕሮጀክት'ውን ኣብ ዘፈረ ሓለዋ ጥዕና ልምዓት ኃይሊ ሰብ ብምጥንኻር ኣብ ኢትዮጵያ ውፅዒት ኣወሓሕባ ግልጋሎት ሓላዋ ጥዕና ወይካኣ ኽንኽን ኣዶታትን /ቅድመ ሕርሲ/ወሊድ (neonatal) ንምምሕይያሽ ፈተነ ወይካኣ ፃዕሪ እንዳገበረ ዝርከብ ፕሮጀክት እዩ፡፡

ዕላማ/ትልሚ

እዚ ፕሮጀክት እዚ ፡- ኣብ ክልላት ትግራይን ኣማሓራን ኣብዝርቡ ትካላት ሓለዋ ጥዕና ዝሰርሑ "መዋላድቲ" ኣብ መደብ ግልጋሎታት ሕማም ሕርሲን ፣ ሕክምና ሕርሲን/ወሊድ Intrapartum (ከይዲ ሕክምና ሕርሲ) ግቡዕ ስራሖምን ኃላፍነቶምን ኣብ ምፍፃም ከይዲ ንዘመዝግብዎ ኩነታ ኣፈፃፀማ ስራሕ ንምርዳዕ ዝተለመ ዕላማ ዘለዎ ፕሮጀክት እዩ፡፡

ከይዲታት ኣፈፃፀማ እቲ መፅናእቲ

- **ተግባራት**

ኣብዚ ምፅናዕቲ እዚ ትሳተፉ/ፉ እንድሕር ኮይንክን/ኩም ፡ ኣብ ከይዲ ሕክምና /ግልጋሎት ሕማም ሕርሲን ፣ ሕክምና ሕርሲን/ወሊድ intrapartum (ከይዲ ሕክምና ሕርሲን) ንዝሕልወክን/ኩም ኣፈጻጽማ ስራሕ ብምትዕዛብ ወይካኣ ብምግምጋም ብውልቂ ቃል-ሕቶ ክንግብረልክን/ኩም ኢና፡፡ እዚ ቃል-ሕቶ እዚ ፡ ብዝኣባ፡- (1) ፍልጠትክን/ኩምን፣ (2) ኣፈጻጽማ ስራሕክን/ኩምን ፣(3) ዝትዓዘብክንኦም/ኩምዎ ኩነታት ጠመተ-ኣፈጻጽማ ስራሕን (4) ማሕበራዊን ዲሞግራፊያዊን ከሙውን ሞያዊ ተሞክሮን ዝጥምት ይከውን፡፡

ዝድለ ጊዜ

እዚ ከይዲ ኣወሓሕባ መረጋገፂ ስምምዕ ተሳትፎ 10 ደቓይቕ ንዝኣክል ጊዜ ዝፀንሕ ኮይኑ ፡ እቲ ከይዲ ቃል-ሕቶ ድማ 30 ደቓይቕ ንዝኣክል ጊዜ ይዝፀንሕ እዩ፡፡ኮይኑ ግና ነቲ ቃል-ሕቶ ንኣክን/ኩም ኣብዝምቸው ካልዕ ጊዜ ንምክያድ ካልእ ቀጸሮ ክንሕዝ ንክዕል ኢና፡፡ይኩን እምበር፣ኣብ ልዕሊ ኣፈጻጽማ ስራሕኩም ዝካየድ ቀጥተኛ "ምልከታ" /direct observation/ ሓንቲ ጥንስቲ ክሳብ ትወልደሉ እዋን ዘዝቅጽል ኮይኑ እቲ intrapartum (ከይዲ ሕክምና ሕርሲ) ክሳብ ዝወዳኣ 6 ሰዓት ንዝኣከል ጊዜ ክፅንሕ ይክዕል፡፡

ምሽጥራዊነት ሓበሬታ ፡

እቲ ቃል-ሕቶ ካልዕ ሰብ ኣብዘይርከበሉ ቦታ ብወልቂ ዘካየድ ይከውን፡፡ ኣብ እዋን እዚ ቃ-ምሕታተ እዚ ዘሰምዓና ወይካአ ዝርዕየና ካልዕ ሰብ ኤሕልውን፡፡ኣብዝኾነ ይኹን ቦታ

IRB #6118
Exempt Determination Date: 04Dec14
Version No.: 1

ሽምክን/ኩም ኣይፃሓፍን፡፡ ድሕሪ እቲ ቃል-ምሕታት ነቲ ሰነድ ቃል-መሕተቲ በሰም ኣብዝተኣሸገ ፓስታ/ኤንቨሎፕ ዘቅምጦ ይከወን ፡፡ ብዚ መሰረት'ውን እቲ ሓበሬታ ምሽጥራዊነቱ ተሓሊው ይተሓዝ ማለት እዩ፡፡ ኣብዚ ትካል እዚ ዝርከብ ወይካአ ዝሰርሕ ዝኾነ ይኹን ሰብ ብኣና ኣቢሉ ንዝርከብ ዝኾነ ይኹን ሓበሬታ ንክርዕይ ወይካኣ ንክረክብ ኣይግባርን/ኣይፍቀደሉን፡፡

እብ ክልል ኣምሓራ ከተማ ባሕር ዳር/ ኣብ ክልል ተግራይ ከተማ መቀለ ብዝርከቡ ኣብያቶ ፅሕፈት ጀፕያጎ ንዝተመልኡ/ንዝምልኡ ሰነዳት መሕተቲ-ቃል/Questionnaires/ ብዝምልከት ዝእከቡ/ዝተኣከቡ ሓበሬታት ብጉጅለ መፅናዕቲና ኣቢሎም ኣብ ኮምፒውተር ዝምዝገቡ ይከውን፡፡ ስለዚ፣ እቲ ናይ መወዳእታ ፅብፃብ ካብ ዘኮነ ይኩን ውልቀሰብ ኪዒላ ወይካኣ ትካል ክንክን ወይካኣ ሓለዋ ጥዕና ዝዕከብ/ዝርከብ ሓበሬታ ኣይግለፀሉን፡፡

ስግዓት/ዘይጥዕም ነገር ኣብዘጋጥመክን/ኩም እዋን፡

ኣብቲ ከይዲ "ምልከታ" ዝእከቡ ሓበሬታታት ብምሽጥር እነድሕር ዘይታሓዙ ኮይኖም ስራሕኩም ወይካኣ ትረክብዎም ጥቅማ ጥቅሚታት ንሓደጋ ክቃልዑ እዮም ዝብል ስግዓት/ጭንቀት ክሓድረኩም ይክዕል ይኽውን፡፡ ኮይኑ ግና እዚ መፅናዕቲ እዚ ኣብዚ ትካል እዚ ናይዝሰርሑ ኪዒላታት ስራሕ ብዝኮን ይኩን መንገዲ ኣይትንክፍን ወይካኣ ኣይጎድዕን፡፡ ብኣካትኩም ዝዋሓቡ መልሲታት ንዝኮነ ይኩን ካልዕ ሰብ/ኣካል ኣይግለፁን፡፡

ረብሓታት/ጥቅሚታት እቲ መጽናዕቲ

መዋለድቲ ዝረክብዎም ረብሓታት/ጥቅሚታ ፡-

- ካብዚ መፅናዕቲ እዚ ቀጥተኛ ረብሓ /ጥቅሚ ኣይትረክቡን ፡፡
- እዚ መፅናዕቲ እዚ ኣብ ከይዲ ምምሕያሽ ፅሬት ግልጋሎት ሓገዝ ክገብር ከምዝክዕል እንትፈልጡ ትረክብዎ ዝክዕል ፅግበት ይሕልው፡፡

አብ ድሌት ዝተደረኽ ተሳትፎ

ክፍሊት የብሉን ፡

እስካትኩም ኮነ እቲ ትሰርሑሉ ትካል ሓለዋ ጥዕና ዝኾነ ይኹን ክፍሊት ወይካኣ ካልዕ ጥቕማ ጥቕሚ አይትረክቡን/አይረክብን ወይካኣ አይዋሓበኩምን/አይዋሓቦን፡፡

አብ ድሌት ዝተደረኽ ንምኳኑ

ናይ ምስታፍን ዘይምስታፍን መሰል ወይካኣ ናፅነት ዘለክን/ኩም እንትኸውን፣ ንምምላስ ንዘይትደልይዎም ሕቶታት መልሲ ንምሓብ አይግደዳን/ዱን፡፡ ብተሳትፎ እንድሕር ተስማሚዕክን/ኩም አብቲ ከይዲ መፅአናዕቲ አብ ዝኾነ ይኹን እዋን ሓሳብክን/ኩም ለዊጥክን/ኩም ነቲ ተሳትፎ ምቁራፅ ትክዕላ/ሉ፡፡ በቲ ተሳትፎ እንድሕር ዘይትሰማምዓ/ዑ ኮይንክን/ኩም ግና፣ ተቋውሞኽን/ኩም አብዚ ወይካኣ አብ ዝኮነ ይኩን ካለዕ ትካል ንትዋፈሩሉ ስራሕ አይትንክፍን ወይካኣ አየተኻናቅፍን፡፡

ንተወሳኺ ሓበሬታ ወይካኣ ሕቶ እንትሕልወክን/ኩም ወይካኣ ዝኾነ ይኹን ፀገም እንተጋጥመክን/ኩም ደዊልክን/ኩም ክተዛርብዎ ዝግባዕ አካል፡-

ንዞም ዝሰዕቡ ስራሕ መካየድቲ መፅናዕቲ ደዊልክን/ኩም ክተዛርቡ ትክዕላ/ሉ፡-

1. ደሳለኝ አደሜ
   ኦፊሰር ክልላዊ ክትትልን ግምገማን
   ቁፅሪ ስልኪ ፡- 251-58 2208083
   ኢሜል
   Desalegn.Ademie@jhpiego.org
   ጆፒያጎ ኢትዮጵያ
   ባህርዳር ክልል አምሓር
1. ምሩፅ ጎሹ
   ስራሕ መካየዲ ክልላዊ ፕሮግራም
   ጆፒያጎ ክልላዊ ቤት ፅሕፈት ትግራይ
   ቁፅሪ ስልኪ ፡- +251 344429212
   ቁፅሪ ሞባይል፡- +251 911276863
   ኢሜል ፡ Miruts.Goshu@jhiego.org
   መቐለ ክልል ትግራይ

- እቲ መፅናዕቲ ንክጅመር ዝወሓብ ፍቃድ ፡-

ነቲ መፅናዕቲ ክጅምር ትፈቅዳልይ ዶ?

ይስማማዕ [ ]　　　　ይቃወም [ ]

[እተን ተሳታፊ እንደሕር ተቃዊመን ፣ ነቲ ተቃውሞ ብፅሑፍ ብምስፋር ናብ ቀፃላይ ተሳታፊ መፅናዕቲ ሰገሩ]

[እተን ተሳታፊ እንድሕር ተስማሚኺን ግና]

አብ መፅናዕትና ንምስታፍ ድሌቱክን ስለዝገልፅክን ነመስገን፡፡ ስለዚ አነ ወይካኣ ክልኦት መሳርሕተይ ንተካይድዎ ምክክር ሕክምና ንምትዕዛብ ክንፅበይና፡፡
