# Supplementary material for: Assessing the competence of midwives to provide care during labor, childbirth and the immediate postpartum period – A cross sectional study in Tigray region, Ethiopia
Source: PLoS One. 2018 Oct 31;13(10):e0206414. doi: 10.1371/journal.pone.0206414 (PMC6209306; doi:10.1371/journal.pone.0206414)
Supplement: S2 File — (PDF) [file pone.0206414.s002.pdf]

IRB #6118
Exempt Determination Date: 04Dec14
Version No.: 1

## RECRUITMENT & ORAL CONSENT SCRIPT #2

## TO BE READ ALLOUD TO CLIENT (WOMAN IN LABOR AND DELIVERY)

## (TIGRIGNA LANGUAGE TRANSALTION)

RECRUITMENT SCRIPT

## ፅሑፍ ምልመላን ናይ ቃል መረጋገፂ ስምምዕ ተሳትፍ ቁፅሪ 2

## ንተገልገልቲ ( ማለትም፡-ኣብ ከይዲ ሕማም ሕርሲን ሕክምና ሕርሲን/ወሊድን ንዝርከባ ደቂኣንስተኦ) "ሓፍ" ብዝበለ ድምፂ ዝንበብ

## ፅሑፍ ምልመላ ምልከታ

ከመይ ቀኒኽን፡ ሽመይ _____________ ይብሓል፡፡ ብዝኣባ ዝኾነ መፅናዕቲ ምሳኩም ክዘራረብ ዲሊየ ኤረ፡፡

ኣብ ፕሮጀክት ሓላዋ ጥዕና መምርሒ ምልማዕ ኃይሊሰብ ከም "መማኽሪ" ኮይነ ዝሰርሕ እንተኸወን፡ እዚ መፅናዕቲ እዚ "መዋለድቲ" ኣብ ሰራሖም ንዝሕልዎም ኩነታ ኣፈፃፀማ ብዝሐሸ መንገዲ ንምርዳእ ወይካአ ንምግምጋም ተሓሲቡ ምስ ክልላዊ ቢሮ ሓለዋ ጥዕና (ምምሕድዳር ክልል ኣምሐራን) ክልላዊ ቢሮ ሓለዋ ጥዕና (ምምሕድዳር ክልል ትግራይን) ብምትሕብባር ዝተካየደ/ዝተዳለወ መፅናዕቲ እዩ፡፡

‹‹ብምዃኑ እውን፡ ግልጋሎት "ሕማም ሕርሲን" "ሕክምና ሕርሲን" /ወሊድን ንምርካብ ናብዚ ትካል እዚ ዝመፅኡ ደቂኣንስቲኦ ኣብዚ መፅናዕቲ እዚ ንክሳተፉልና ንእድም፡፡"

"ምሳኽን ንቁሩብ ደቓይቅ ክዘራረብ ስለዝደለኹ፡ ሕጂ ሓንሳብ ጊዜ ወሲድክን ክትድምፃኒ ፍቓደኛ ዲኽን?"

["ኣይኮንኩን" እንድሕር ኮይኑ] "ፅቡቅ ፣ ንዝሓባኒ ጊዜ የመስገን፤ ድሓን ዋዓላ"

[ "እው" እንድሕር ኮይኑ]

እቲ መቕረባይ ቃል-ሕቶ ነቲ ተቃውሞ ኣብቲ መዝገብ ምልመላ ብፅሑፍ ብምስፋር፣ ሕቶኡ ሒዙ ኣብ ሕማም-ሕርሲን ከይዲ ሕክማና ሕረሲን/ወሊድን ናብዝርከባ ቀፃሊት/ካልዕ ወላዲት ዌካኣ ጥንስቲ ይሰግር ::

IRB #6118
Exempt Determination Date: 04Dec14
Version No.: 1

[“እወ” እንድሕር ኮይኑ] “የቀንየለይ፣ እምበአርከስ ብዝኣባ ክንስርሕ ዝተለምናዮም ነግራት ተወሳኺ ሓበሬታ ክሕበክን”

“ዝሰልጠኑ ኪዒላታት “ምልከታ/ትዕዝብቲ ( ማለትም አነ ) ምስ ብሰራሕተኛ ኽንኽን ወይካኣ ሓለዋ ጥዕና ንዝዋሓበክን  ከይዲ ሕማም ሕርሲን ሕክምና ሕርሲን/ወሊድን ንክዕዘብ ክትፈቅዳለይ ክሓተክን ኮይኑ፣ ኩሎም ኪዓላታት ምልከታ/ትዕዝብቲ ብዓንፃር አወሓሕባ ግልጋሎት ኽንኽን ወይካኣ ሓለዋ ጥዕና  አብ እዋን ከይዲ ምክክር ሕክምና ብዝኣባ ዝካየድ ስነ-ስርዓት አፈፃፀማን ምይይጥን እኹል ተሞክሮን ፍልጠትን ዘለዎም “ስልጡን” ኪዒላታት ኽንኽን ወይካኣ ሓለዋ ጥዕና ከምዝኾኑ ክነረጋግፅ ንፈቱ፡፡ ብምኟኑ’ውን፣ አብ እዋን ከይዲ አወሓሕባ ግልጋሎት ሕማም ሕርሲን ሕክምና ሕርሲን/ወሊድን እቲ ገምጋሚ መዝከርታ እንዳመዝገበ/እንዳሐዘ ነቲ ከይዲ ምክክር/ምይይጥ ሱቕ ኢሉ ዝከታተል ይኸውን፡፡”

“ሽምክን ወይካአ ሽም ዘመድንክን ዘይምዝገብ እንተኸውን፣ ብዝኣባ እዚ ዋኒን እዚ ዝርከቡ ሐበሬታት ብሙሉኦም ብምሽጥር ዝተሓዙ ይኸውን፡፡”

“አብቲ መጽናዕቲ ናይ ግድን ክትሳተፋ አይትግደዳን፡፡ ንምስታፍ ብዘይምድላይኽን ዘመድክን ሎሚ ወይካኣ ንቀፃላይ ንዝረክብዎ ግልጋሎት ኽንኽን ወይካኣ ሐለዋ ጥዕና አይትንክፍን ወይካኣ አይተኸናቅፍን፡፡ ኮይኑ ግና አብዚ መፅናዕቲ እዚ እንተተሳቲፍክን አብዚ ትካል እዚ ናይ ዝዋሓብ ግልጋሎት ምኽንኻን ወይካኣ ሐለዋ ጥዕና ፅሬት አብ ምምሕይያሽ ከይዲ ናይ ባዕሉ ሓገዝ ከምዝገብር ክንሕብረክን ንፈቱ፡፡”

“ብዝኣባ እዚ መፅናዕቲ እዚ ዝርዝር ሓበሬታ ናይምፍላጥ ድሌት አለክን ዶ?”

[“የብለይን” እንድሕር ኮይኑ] “ፅቡቕ ፣ ንዝሓብክና ጊዜ ነመስገን ፤ድሓን ዋዕላ!”

እቲ መቕረባይ ቃል-ሕቶ ነቲ ተቃውሞን ዓይነት ተገልጋሊትን አብ መዝገብ ምልመላ ተሓኪምቲ/ተገልገልቲ ብፅሑፍ የሰፍር፡፡

[“እወ” እንድሕር ኮይኑ] “ፅቡቅ ፣ እምበአርከስ ብዝኣባ እዚ መፅናዕቲ እዚ ዝርዝር ሐበሬታ ክሕበክን፡፡ አብዚ መፅናዕቲ እዚ ንምስታፍ እንድሕር ተስማሚዕክን ድሌትክን ክተፍልጣኒ ትክዕላ”

[ብዚ መሰረት‘ውን እቲ መቕረባይ ቃል -ሕቶ ቅልጥፍ ኢሉ ወይካአ ብእዋኑ ነቲ ከይዲ አፈፃፅማ (ናይ ቃል) መረጋገፂ ስምምዕ ተሳትፎ  አብ ቀፃሊ ገፅ ምድላው/ምምላዕ ይጀምር]

## ORAL CONSENT SCRIPT #2

## ፅሑፍ ምልመላን ናይ ቃል መረጋገፂ ስምምዕ ተሳትፍ ቁፅሪ  2

IRB #6118
Exempt Determination Date: 04Dec14
Version No.: 1

## ንተገልገልቲ ( ማለትም፦አብ ከይዲ ሕማም ሕርሲን ሕክምና ሕርሲን/ወሊድን ንዝርከባ ደቂአንስተኦ) "ሓፍ" ብዝበለ ድምፂ ዝንበብ

**ዓርስቲ መፅናዕቲ** ፡ አብ ኢትዮጵያ ክልላት ትግራይን አምሓራን አብዝርከቡ ትካላት ሓለዋ ጥዕና ንዝሰርሑ "መዋለድቲ" ዝምላዕ ሰነድ መገምገሚ ብቅዓት

**ዋና ኪዒላ መፅናዕቲ/መርማሪ** ፦ ዶ/ር ያንግ ሚ ኪም

**ቁፅሪ አይአርቢ** ፡ 6118

## ዕላማ

አርስክን አብዚ መፅናዕቲ እዚ ንክትሳተፉ ስለዝተኣደምክን፤ ብዝኣባ እቲ መፅናዕቲ ቁሩብ መብርሒ ክሕበክን ይፈቱ፡፡

## ነቲ መፅናዕቲ ዘካይዶ አካል

እዚ መፅናዕቲ እዚ ዝካየድ ዘሎ አብ ኢትዮጵያ ብዝርከብ ፕሮጀክት ሓላዋ ጥዕና መምርሒ ምልማዕ ኃይሊ ሰብ እንተኸውን ፤ እዚ ፕሮጀክት'ውን አብ ዘፈረ ሓለዋ ጥዕና ልምዓት ኃይሊ ሰብ ብምጥንኻር አብ ኢትዮጵያ ውፅዒት አወሓሕባ ግልጋሎት ሓላዋ ጥዕና ወይካኣ ኽንኽን አዶታትን /ቅድመ ሕርሲ/ወሊድ (neonatal) ንምምሕይያሽ ፈተነ ወይካኣ ፃዕሪ እንዳገበረ ዝርከብ ፕሮጀክት እዩ፡፡

## ዕላማ/ትልሚ

እዚ ፕሮጀክት እዚ ፦ አብ ክልላት ትግራይን አማሓራን አብዝርቡ ትካላት ሓለዋ ጥዕና ዝሰርሑ "መዋለድቲ" አብ መደብ ግልጋሎታት ሕማም ሕርሲን ፤ ሕክምና ሕርሲን/ወሊድ Intrapartum (ከይዲ ሕክምና ሕርሲን) ግቡዕ ስራሖምን ኃላፍነቶምን አብ ምፍፃም ከይዲ ንዘመዝግብዎ ኩነታ አፈፃፀማ ስራሕ ንምርዳዕ ዝተለመ ዕላማ ዘለዎ ፕሮጀክት እዩ፡፡

## ከይዲታት አፈፃፀማ እቲ መፅናእቲ

- **ተግባራት**

አብዚ ምፅናዕቲ እዚ ትሳተፉ እንድሕር ኮይንክን ፡ አብ ክይዲ ሕክምና /ግልጋሎት ሕማም ሕርሲን ፣ ሕክምና ሕርሲን/ወሊድ Intrapartum (ክይድ ሕክምና ሕርሲን) ብ “መዋለድቲ” አቢሉ ንዝወሓበክን ክንክን ክንእዝብ/ክንከታተል ኢና።

- **ዝድለ ጊዜ**

እዚ ክይዲ አወሓሕባ መረጋገፂ ስምምዕ ተሳትፎ 10 ደቓይቕ ንዝኣክል ጊዜ ዝፀንሕ ኮይኑ ፡ አብ ልዕሊ ገምጋም ክይዲ አወሓሕባ ኽንኽን “መዋለድቲ” ዝግበር ክይዲ ቀጥተኛ “ምልከታ” /direct observation/ ድማ ዝግበራልክን intrapartum (ክይዲ ሕክምና ሕርሲ) ክሳብ ዝወዳኣ 6-8 ሰዓት ንዝኣከል ጊዜ ክፅንሕ ይክዕል።

## ምሽጥራዊነት ሐበሬታ ፡

ሽምክን ወይካኣ ሽም ትወልዱሉ ትካል ክንክን ወይካኣ ሐለዋ ጥዕና አይፃሓፍን። ንዓክን ዝምልከቱ ውልቃዊ ሓበሬታታት ብፅሑፍ አይምዝገቡን ወይካኣ ብዝኣባ ናኣትክን ሕማም-ሕርስን ኩነታተታት ክይዲ ሕክምና ሕርስን/ወሊድን ንዝኮን ይኩን ካልእ ሰብ አይግለፅን ።

## ስግዓት/ዘይጥዕም ነገር አብዘጋጥመክን እዋን፡

አብቲ ክይዲ “ምልከታ” ኪዒላታት ምኽንኻን ወይካአ ሓለዋ ጥዕና ጽሬት ዘለዎም ግልጋሎታት አይሕቡኒን ዝብል ስግዓት/ጭንቀት ክስማአክን ይክዕል ይኸውን። ኮይኑ ግና እዚ መፅናዕቲ እዚ አብዚ ትካል እዚ ናይዝሰርሑ ኪዒላታት ስራሕ ወይከኣ ንኣኽን ዝዋሓበክን ግልጋሎት ምኽንኻን ወይካአ ሐለዋ ጥዕና ኣይትንክፍን ወይካኣ አይጎድዕን።

ሽምክን ሽም ዘመድክንን ዘይምዝገብ እንትኸውን ነቶም ንእክቦም ሓበሬታት’ውን ንዝኾነ ይኹን ካልዕ ሰብ ኣይንሕብን ወይካኣ አይነርዕይን።

## ረብሓታት/ጥቅሚታት እቲ መጽናዕቲ

ተገልገልቲ ዝረክብዎም ረብሓታት ፡

- ካብዚ መፅናዕቲ እዚ ቀጥተኛ ረብሓ /ጥቅሚ ኣይትረክባን፡፡
- እዚ መፅናዕቲ እዚ ኣብ ከይዲ ምምሕያሽ ፅሬት ግልጋሎት ሓገዝ ክገብር ከምዝክዕል እንትፈልጡ ትረክብዎ ዝክዕል ፅግበት ይሕልው፡፡

## ኣብ ድሌት ዝተደረኸ ተሳትፎ

**ክፍሊት የብሉን ፡**

ኣብዚ መፅናዕቲ እዚ ብምስታፍክን ዝኾነ ይኹን ክፍሊት ወይካኣ ካልዕ ዓይነት ጥቕማ ጥቕሚ ኣይትረክባን ወይካኣ ኣይዋሓበክንን፡፡

## ኣብ ድሌት ዝተደረኸ ንምኧኑ

ናይ ምስታፍን ዘይምስታፍን መሰል ወይካኣ ናፅነት ዘለክን ዕንትኸውን፣ ብተሳትፎ እንድሕር ተስማሚዕክን ኣብቲ ከይዲ ምክክር ሕክምና ኣብ ዝኾነ ይኹን እዋን ሓሳብክን ለዊጥክን ተሳትፎክን ክተቋርፃ ትክዕላ ኢኽን፡፡ ብቲ ተሳትፎ እንድሕር ዘይትሰማምዓ ኮይንክን ግና፣ ተቋውሞኽን ኣብዚ ትካል ወይካኣ ኣብ ዝኮን ይኩን ከልዕ ትካላት ንዝዋሓበክን ግልጋሎት ምኽንኻን ወይካኣ ሐለዋ ጥዕና ኣይትንክፍን ወይካኣ ኣየተኣናቅፍን፡፡

IRB #6118
Exempt Determination Date: 04Dec14
Version No.: 1

***ንተወሳኺ ሓበሬታ ወይካኣ ሕቶ እንትሕልወክን ወይካኣ ዝኾነ ይኹን ፀገም እንተጋጥመክን ደዊልክን ክተዛርብዎ ዝግባዕ አካል፡-***

| |
|---|
| ንዞም ዝሰዕቡ ስራሕ መከየድቲ መፅናዕቲ ደዊልክን ክተዛርባ ትክዕላ፡- <br><br> 1. ደሳለኝ አደሜ <br> ኦፊሰር ክልላዊ ክትትልን ግምገማን <br> ቁፅሪ ስልኪ ፡- 251-58 2208083 <br> ኢሜል <br> Desalegn.Ademie@jhpiego.org <br> ጂፒያጎ ኢትዮጵያ <br> ባህርዳር  ክልል አምሓር <br> 2. ምሩፅ ጎሹ <br> ስራሕ መካየዲ ክልላዊ ፕሮግራም <br> ጂፒያጎ ክልላዊ ቤት ፅሕፈት ትግራይ <br> ቁፅሪ ስልኪ ፡- +251 344429212 <br> ቁፅሪ ሞባይል፡- +251 911276863 <br> ኢሜል ፡ Miruts.Goshu@jhiego.org <br> መቐለ ክልል ትግራይ |

- እቲ መፅናዕቲ ንክጅመር ዝወሓብ ፍቃድ ፡-

ነቲ መፅናዕቲ ክጅምር ትፈቅዳልይ ዶ?

ይስማማዕ [ ]          ይቃወም [ ]

[እተን ተሳታፊ እንደሕር ተቃዊመን ፣ ነቲ ተቃውሞ ብፅሑፍ ብምስፋር ናብ ቀፃላይ ተሳታፊ መፅናዕቲ ሰገሩ]

[ እተን ተሳታፊ እንድሕር ተስማሚኸን ግና]

አብ መፅናዕትና ንምስታፍ ድሌቱክን ስለዝገልፅክን ነመስገን፡፡ ስለዚ አነ ወይካኣ ክልኦት መሳርሕተይ ንተካይድዎ ምክክር ሕክምና ንምትዕዛብ ክንፀበይና፡፡
