# Supplementary material for: Assessing the competence of midwives to provide care during labor, childbirth and the immediate postpartum period – A cross sectional study in Tigray region, Ethiopia
Source: PLoS One. 2018 Oct 31;13(10):e0206414. doi: 10.1371/journal.pone.0206414 (PMC6209306; doi:10.1371/journal.pone.0206414)
Supplement: S3 File — (PDF) [file pone.0206414.s003.pdf]

RECRUITMENT & ORAL CONSENT SCRIPT #1
TO BE READ ALOUD TO MIDWIVES

Recruitment Script

Hello, my name is_______________. I'd like to speak with you about a study.

I am a consultant for the Human Resources for Health Project. This study is conducted in collaboration with Amhara regional health bureau (for Amhara region) and Tigray regional health Bureau (for Tigray region) to better understand performances at your job, including your knowledge, performance and perceived understanding the factors affecting your performance

"I'd like to speak with you for a few minutes. Do you have a few minutes to listen now?" [IF NO] "May I

talk to you at another time?"

[IF NO] "Okay, thank you for your time. Have a good day!"

INTERVIEWER NOTES REFUSAL IN RECRUITMENT LOG.

[IF YES] "Thank you. Let's arrange another time to talk that is more convenient for you." INTERVIEWER ARRANGES A MEETING WITH THE PROVIDER AND FOLLOWS UP. AT THAT MEETING, INTERVIEWER BEGINS WITH THE NEXT STEP BELOW [IF YES]

[IF YES]: "Thank you! Let me tell you more about what we would like to do."

"We will observe your performance during labour, delivery and intrapartum care. We will also ask you some questions about your work at this facility, including your knowledge, performance and perceived understanding the factors affecting your performance, and availability of equipment and infrastructures.

"We will <u>not</u> write down your name anywhere. We will <u>not</u> show the data to <u>anyone</u> at this or other health facilities. All data will be kept confidential."

"You do not have to participate. If you do not want to participate, it will <u>not</u> affect your job or benefits here. "Are you interested in learning more about this study?"

[IF NO]: "Okay, thank you for your time. Have a good day!"

INTERVIEWER NOTES REFUSAL ON THE RECRUITMENT LOG.

[IF YES]: "Okay, I will tell you more about the study. Then you can then tell me whether you agree to participate."

[IMMEDIATELY INTERVIEWER CONDUCTS ORAL CONSENT PROCESS ON NEXT PAGE.]

(Once approved, IRB logo goes here)	***IRB Office Use Only:*** Approval date: Approved consent version No.:

:

ORAL CONSENT SCRIPT # 1
TO BE READ TO <u>MIDWIFE</u>

STUDY TITLE: PERFORMANCE ASSESSMENT OF MIDWIVES IN PROVISION OF CARE DURING LABOR, CHILDBIRTH AND IMMEDIATE POSTPARTUM PERIOD IN TIGRAY AND AMHARA REGIONS, ETHIOPIA

Principal Investigator: Dr. Young Mi Kim

IRB No.: 6118

PURPOSE

You are being invited to take part in a research study. Let me explain a little about the study.

Who Is Conducting the Study

The Human Resources for Health Project in Ethiopia is carrying out this study. The project is trying to improve maternal and neonatal health outcomes in Ethiopia by strengthening human resources for health.

Purpose/Aims

The aim of this study is to understand midwives performance to manage labor, delivery and intrapartum care in the health facilities of Amhara region and Tigray region

PROCEDURES

What We Will Do

If you participate in this study, we will observe your performance and interview you privately about: (1) your knowledge (2) your performance (3) perceived performance factors and (4) socio- demographic information and professional experience.

Time Needed

This consent process takes about 10 minutes, and the interview will take about 30 minutes. We can schedule the interview for a time that is convenient for you. But, direct observation of your performance will take until a woman give birth and 6 hours of postpartum

:

Confidentiality of Data

The interview will take place in a private place. No one else will be present. No one else will see or hear you during the interview.

We will not record your name. After the interview, I will put your questionnaire in a sealed envelope. We will keep your answers confidential. We will not show them to anyone at this or any other health facility.

When we return to the completed questionnaire the Jhpiego office in Bahir Dar for Amhara region/Mekele for Tigray region, our study team will enter the data in a computer. The final report will not present information from any individual provider or any one health facility.

RISKS/DISCOMFORTS

You may worry that your job or benefits may be at risk if the data are not kept confidential. However, the study will not affect your job in any way. We will take several measures to keep all data confidential. Your name and ID number will not be written down anywhere. We will not show your answers to anyone.

BENEFITS

Benefits to you

- You will receive no direct benefit from the study.
- You may get some satisfaction from knowing that the study may help improve the quality of performance

VOLUNTARY PARTICIPATION

No Payment

You will not receive any payment or compensation. The health facility also will not receive any payment.

Voluntary

You are free to participate or not. You do not have to answer any questions that you do not want to. If you agree to participate, you can change your mind and stop participating at any time. If you decide not to participate, this will not affect your job at this health facility or another facility in any way.

(Once approved, IRB logo goes here)	**IRB Office Use Only:**<br>Approval date:<br>Approved consent version No.:

:

Who should you call for more information, or if you have questions or problems?

Call the Study Managers

1. *Desalegn Ademie*

Regional Monitoring and Evaluation officer
Tel: 251-58 220 8083
Email: Desalegn.Ademie @jhpiego .org
Jhpiego Ethiopia
Bahir Dar, Amhara Region

…………………………………………………………………………………..

2. **Miruts Goshu**

Regional Program manager Jhpiego, Tigray Regional Office
Tele: +251 344 419 212
Mobile: +251 911 176 863
Email: Miruts.Goshu@jhpiego.org
Mekele, Tigray region

PERMISSION TO PROCEED

May I have your permission to proceed with the study?

Agree [ ] Refused [ ]

If you agree, we can schedule the interview.

[IF THE PARTICIPANT REFUSES, NOTE THE REFUSAL AND GO TO THE NEXT POTENTIAL STUDY PARTICIPANT.]

[IF THE PARTICIPANT AGREES]

Thank you for your consideration. Let's schedule a time to conduct the interview that is convenient for you.
