# Supplementary material for: Assessing the competence of midwives to provide care during labor, childbirth and the immediate postpartum period – A cross sectional study in Tigray region, Ethiopia
Source: PLoS One. 2018 Oct 31;13(10):e0206414. doi: 10.1371/journal.pone.0206414 (PMC6209306; doi:10.1371/journal.pone.0206414)
Supplement: S4 File — (PDF) [file pone.0206414.s004.pdf]

Exempt Determination Date: December 4,2014
Consent Version No.: 1
PI Name: Young Mi Kim
IRB No.: 6118

RECRUITMENT & ORAL CONSENT
SCRIPT # 2

TO BE READ ALOUD TO <u>CLIENT (WOMAN IN LABOR AND DELIVERY)</u>

Recruitment Script for Observation

Hello, my name is__________ . I'd like to speak with you about a study.

I am a consultant for the Human Resources for Health Project. This study is conducted in collaboration with Amhara regional health bureau (for Amhara region) and Tigray regional health Bureau (for Tigray region) to better understand performances of midwives at their jobs.

"We'd like to invite women who are coming for labor and delivery services.

"I'd like to speak with you for a few minutes. Do you have a few minutes to listen now? "
[IF NO]: "Okay, thank you for your time. Have a good day!"
INTERVIEWER NOTES REFUSAL, ASK THE NEXT LABOR AND DELIVERY WOMAM

[IF YES]: "Thank you! Let me tell you more about what we would like to do."

"We will ask you to allow a trained observer/assessor (me) to observe your labor, delivery and intrapartum care services given by the provider. All assessors are trained in healthcare providers who are familiar with the procedures and discussions that take place during medical consultations, labor and delivery services. During the labor and delivery services, the assessor will take notes and remain silent."

"We will <u>no</u>t write down your name and not ask you follow up questions. All information will be kept confidential."

"You do not have to participate. If you do not want to participate, it will <u>not</u> affect the health care you receive today or in the future. By participating, you can help improve the quality of health care offered in this facility or another facility in the region. "

"Are you interested in learning more details about this study?"

[IF NO]: "Okay, thank you for your time. Have a good day!"
INTERVIEWER NOTES REFUSAL, ASK THE NEXT LABOR AND DELIVERY WOMEN.

[IF YES]: "Okay, I will tell you more about the study. You can then tell me if you agree to participate."
[IMMEDIATELY INTERVIEWER CONDUCTS ORAL CONSENT PROCESS ON NEXT PAGE.]

ORAL CONSENT SCRIPT #2 FOR OBSERVATION

TO BE READ ALOUD TO CLIENT (WOMAN IN LABOR AND DELIVERY)

Study Title: Assessment of midwives performance to manage labor and delivery in the health facilities of Amhara and Tigray Regions, Ethiopia
Principal Investigator: Dr. Young Mi Kim

IRB No.: 6 1 1 8

PURPOSE

You are being invited to take part in a research study. Let me explain a little about the study.

Who Is Conducting the Study

The Human Resources for Health Project in Ethiopia is carrying out this study. The project is trying to improve maternal and neonatal health outcomes in Ethiopia by strengthening human resources for health.

Purpose/Aims

The aim of this study is to understand midwives performance to manage labor, delivery and intrapartum care in the health facilities of Amhara region and Tigray region.

PROCEDURES

What We Will Do

If you participate in this study, we will observe you while midwives are providing care to you during labor, delivery and intrapartum.

Time Needed

This consent process takes about 10 minutes. Direct observation of midwives performance may take 6-8 hours until your intrapartum care is completed.

Confidentiality of Data

We will not record your name or the name of health facility that you give birth. We will not record your personal information or tell to any other person about your labor and delivery situation.

RISKS/DISCOMFORTS

You may worry that the provider will not give you quality services during observation process. However, the study will not affect provider's job and your health care service in this facility.

BENEFITS

Benefits to you

- You will receive no direct benefit from the study.
- You may get some satisfaction from knowing that the study may help improve the quality of service

VOLUNTARY PARTICIPATION

No Payment

You will not receive any payment or compensation. The health facility also will not receive any payment.

Voluntary

You are free to participate or not. If you agree to participate, you can change your mind and stop participating at any time. If you decide not to participate, this will not affect your health care services in this health facility or another facility in any way.

Who should you call for more information, or if you have questions or problems?

Call the Study Managers

1. *Desalegn Ademie*

Regional Monitoring and Evaluation officer
Tel: 251-58 220 8083
Email
Desalegn.Ademie @jhpiego .org
Jhpiego Ethiopia
Bahir Dar, Amhara Region

…………………………………………………………………………………..

2. **Miruts Goshu**

Regional Program manager Jhpiego, Tigray Regional Office
Tele: +251 344 419 212
Mobile: +251 911 176 863
Email: Miruts.Goshu@jhpiego.org
Mekele, Tigray region

:

PERMISSION TO PROCEED

May I have your permission to proceed with the study?

Agree [ ]   Refused [ ]

If you agree, we can observe your labor, delivery and intrapartum care giving services

[IF THE PARTICIPANT REFUSES, NOTE THE REFUSAL AND ASK THE NEXT LABOR AND DELIVERY WOMEN

[IF THE PARTICIPANT AGREES]

Thank you for your consideration. I will wait so that we can observe your labor, delivery and intrapartum care services.
